# In Vivo Fluorescent Melanoma Model: Electroporation Plus Magnetic Hyperthermia Significatively Reduces Tumor Size, Preliminary Results

**DOI:** 10.3390/pharmaceutics18070783

**Published:** 2026-06-26

**Authors:** Andrea Molina-Pineda, Sayma Vizcarra-Ramos, Abel Gutiérrez-Ortega, Adriana Aguilar-Lemarroy, Luis F. Jave-Suárez, Mario E. Cano, Rodolfo Hernández-Gutiérrez

**Affiliations:** 1 Centro de Investigación y Asistencia en Tecnología y Diseño del Estado de Jalisco, A.C. (CIATEJ), Guadalajara 44270, Mexico; andymopi@gmail.com (A.M.-P.); aortega@ciatej.mx (A.G.-O.); 2 Centro de Investigación Biomédica de Occidente (CIBO), División de Inmunología, Instituto Mexicano del Seguro Social (IMSS), Guadalajara 44340, Mexico; sayvimay0510@gmail.com (S.V.-R.); adry.aguilar.lemarroy@gmail.com (A.A.-L.); lfjave@gmail.com (L.F.J.-S.); 3 Centro Universitario de la Ciénega, Universidad de Guadalajara, Avenida Universidad 1115, Ocotlan 47810, Mexico

**Keywords:** electroporation, magnetic hyperthermia, SPIONs, in vivo assays

## Abstract

**Background/Objectives:** Melanoma affects both sexes, and its incidence has increased in recent years. It is currently among the most common types of cancer. Standard chemotherapy, although effective, often lacks selectivity for tumor cells, resulting in dose-limiting side effects. Electrochemotherapy and magnetic hyperthermia have been investigated as innovative biomedical approaches. Electrochemotherapy improves drug delivery by facilitating electroporation, thereby increasing intracellular concentrations of chemotherapeutic agents and reducing associated adverse effects. Furthermore, electroporation enhances sensitivity to magnetic hyperthermia. However, few studies have focused on the combination of electroporation and hyperthermia in melanoma models. This study aimed to evaluate the synergistic effects of intratumoral administration of superparamagnetic iron oxide nanoparticles (SPIONs), electroporation (EP), and magnetic hyperthermia (EHP) on fluorescent melanoma tumors generated with the MV3-GFP cell line. **Methods:** Fluorescent melanoma tumors were generated using the MV3-GFP cell line. Treatments included SPIONs alone, SPIONs combined with hyperthermia, and SPIONs combined with electroporation and hyperthermia. Tumor size was monitored over 21 and 28 days. **Results:** SPIONs alone did not affect tumor growth (665 mm^3^). SPIONs plus hyperthermia reduced tumor size to 126.5 mm^3^ at day 21. The combination of SPIONs, electroporation, and hyperthermia produced a pronounced antitumoral effect, with tumor size decreasing to 95.5 mm^3^ at day 14 and 6.8 mm^3^ at day 21, followed by complete tumor disappearance by day 28. Electroporation significantly enhanced the antitumoral activity of the combined treatment. **Conclusions:** The combination of SPIONs, electroporation, and magnetic hyperthermia shows significant synergistic antitumoral activity in a melanoma model. These findings support further investigation in larger and more comprehensive in vivo studies to better understand the therapeutic potential of these combined approaches.

## 1. Introduction

Malignant melanoma (MM) is the most aggressive type of skin cancer, and arises from melanocytes, which are pigment-producing cells derived from the neuroectoderm with a highly polarized dendritic morphology [1]. Malignant melanoma represents a more dangerous form of skin cancer, characterized by its increasing prevalence globally and resistance to various therapeutic modalities. Melanoma cancer is the 17th most common cancer worldwide and the 22nd leading cause of death in both females and males, with approximately 332,000 new cases and 58,700 deaths estimated in 2022 [2].

To address the main drawbacks of chemotherapy, emerging technologies such as electrochemotherapy increase drug selectivity and effectiveness. This technique, which utilizes electroporation, facilitates the uptake of low-permeability chemotherapeutic drugs such as doxorubicin, bleomycin and cisplatin. It increases the intracellular drug concentration and cytotoxicity while minimizing side effects [3,4]. Electroporation is also used in nanomedicine to deliver nanoparticles into cells [5]. Superparamagnetic iron oxide nanoparticles (SPIONs) represent a promising avenue, leveraging their magnetic properties to induce hyperthermia when exposed to alternating magnetic fields [6]. Magnetic hyperthermia increases the temperature to 40–45 °C and, in addition to inducing tumoral cell death [7,8], can potentially augment the efficacy of chemotherapy by increasing the cellular sensitivity to drugs in vivo and inducing apoptosis [9].

While studies have demonstrated the inhibitory effects of magnetic and laser hyperthermia on the growth of several types of cancer cells in vitro and in vivo [10,11,12,13,14] and its synergy with chemotherapy enhances tumor remission [15], limited research has been conducted on the effects of electroporation in combination with hyperthermia in melanoma cancer models. In this preliminary study, the synergistic effects of electroporation plus hyperthermia in combination with magnetic hyperthermia (which uses SPIONs) on the melanoma MV3-GFP cell line in vitro were assessed.

## 2. Materials and Methods

### 2.1. Cell Culture

The MV3-GFP cell line was obtained from Anticancer, Inc. (San Diego, CA, USA) The cells were cultured in Opti-MEM, supplemented with 10% fetal bovine serum, 100 U Penicillin and 100 µg/mL Streptomycin at 37 °C and 5% carbon dioxide (CO_2_).

### 2.2. Cytotoxicity Assays

#### 2.2.1. MTT

To determine the cytotoxic effect of the SPIONs, an MTT cell viability assay was performed in 96-well plates. A total of 2 × 10^4^ cells/well were seeded and incubated overnight. After monolayer formation, the cells were treated with electroporation, SPIONs alone, SPIONs plus electroporation, SPIONs plus hyperthermia or SPIONs plus electroporation plus hyperthermia. Cell viability was measured 24 h after exposure to the treatments. Next, the Opti-MEM medium was removed, and the cells were washed with PBS. Then, 100 μL of Opti-MEM and 10 μL of 5 mg/mL MTT solution (Sigma-Aldrich, St. Louis, MO, USA) were added and the cells were incubated for 3 h at 37 °C. After the MTT solution was removed, the remaining crystals in the wells were solubilized. The absorbance was determined at a wavelength of 570 nm. Each experiment was repeated at least three times in triplicate.

#### 2.2.2. Electrochemotherapy In Vitro Standardization

A total of 1 × 10^5^ cells/well in a 1 mL final volume were seeded in a 24-well plate and 8 monopolar square wave pulses of 100 µs were delivered immediately at a repetition frequency of 1 Hz by an electroporation power supply (ELECTROvet EZ, Leroy Biotech, Saint-Orens-de-Gameville, France) using a plate-shaped contact electrode (8 mm gap) introduced into each well. For the combination of electroporation, 1 × 10^5^ cells were collected in 500 µL of OPTI-MEM medium, transferred to a 24-well plate and electroporation was applied. After electroporation, 100 µL of cell suspension was seeded per triplicate in a 96-well plate and incubated for 24 h, after which MTT assays were performed.

#### 2.2.3. SPION’s Heating Capability and Cytotoxicity

Magnetic hyperthermia assays were performed using a ferrofluid of superparamagnetic iron oxide nanoparticles (SPIONs) synthesized by the co-precipitation method described by Cervantes et al., 2022 [16]. The optimal concentrations of SPIONs for hyperthermia assays were determined as described by Vizcarra-Ramos et al., 2024 [13].

#### 2.2.4. Magnetic Hyperthermia Standardization In Vitro

The optimal time for magnetic hyperthermia was determined using the MV3-GFP cell line, considering that the irradiation time of the treatment should reduce cell viability by at least 50% [13]. After 24 h, the medium containing the SPIONs was discarded and the cells were washed with D-PBS. Finally, 100 µL of fresh medium was added. Cell viability was measured using the cell proliferation reagent MTT.

#### 2.2.5. Electrochemotherapy Combined with Magnetic Hyperthermia In Vitro

Once the optimal concentration of SPIONs and the optimal time of magnetic hyperthermia treatment were determined, the hyperthermia assay was evaluated in combination with electroporation in the MV3-GFP cell line. Treatments were applied to suspended (trypsinized) cells. For the groups of untreated cells, 3 × 10^4^ cells per well were seeded in a volume of 250 µL into a 48-well plate. A total of 250 µL of the corresponding treatment mixture was added: Opti-MEM medium for the control group or SPIONs at a final concentration of 1 mg/mL. The electroporation variable was added to all the above groups. For this purpose, 9 × 10^4^ cells were collected in 750 µL of Opti-MEM medium and 750 µL of the above treatments were added. Then, with plate-shaped electrodes with an 8 mm gap between them, 8 pulses of monopolar square waves of 100 µs at 1000 V/cm with a repetition frequency of 1 Hz were applied using an electroporator (ELECTROvet EZ, Leroy Biotech). The cells were homogenized and 3 × 10^4^ cells/500 µL were transferred to the 48-well plate used for the control groups and incubated for 24 h. For the experimental groups subjected to hyperthermia, 9 × 10^4^ cells were collected in 250 µL Opti-MEM medium in a 2 mL microtube and 250 µL of the corresponding treatment was added: 1 mg/mL of SPIONs for the hyperthermia group and electroporation plus hyperthermia. For the electroporation groups, the corresponding volume was transferred from the microtube to a 24-well plate and 8 pulses of monopolar square waves of 100 µs at 1000 V/cm with a repetition frequency of 1 Hz were applied using a plate-shape electrode with 8 mm gap between them and an electroporator (ELECTROvet EZ, Leroy Biotech). Then, the pulses were returned to the 2 mL microtube.

All hyperthermia experimental groups were incubated at 37 °C for 20 min, and a dry bath was maintained at 37 °C during the hyperthermia process. Each tube was irradiated with an magnetic field at a heating frequency (f) of 460 kHz and an amplitude (H) of 20 kA/m for 10 min; in the first 5 min, a temperature of 43 °C was reached, and this temperature was maintained for 5 min by modifying the amplitude conditions. The temperature was measured using a fiber optic probe as described by Vizcarra-Ramos et al., 2024 [13].

Once hyperthermia was induced in all the experimental groups, 1 mL of Opti-MEM medium was added to each tube to obtain a final concentration of 1 mg/mL of SPIONs, the cells were homogenized and 3 × 10^4^ cells/500 µL were transferred to a 48-well plate and incubated. After 24 h, the medium supplemented with the SPIONs was discarded, and the cells were washed with D-PBS. Finally, 100 µL of fresh medium was added. Cell viability was measured using the MTT assay (10 µL was added to each well and the mixture was incubated for four hours).

#### 2.2.6. In Vivo Assays

Twelve-week-old immunodeficient nude murine females of the Nu/Nu strain were acquired from Bioterio Morelos (Tlayacapan, Morelos, Mexico)d kept at the Research Bioterium of the Center for Research and Assistance in Technology and Design of the State of Jalisco (CIATEJ). The experiments were conducted in accordance with the guidelines set by the Guide for the Care and Use of Laboratory Animals and the rules established by the Internal Committee for Care and Use of Laboratory Animals of CIATEJ under the approved project number (2023-008A).

The MV3/GFP cells were cultured in Opti-MEM medium and inoculated into the murine models. Cultured cells reaching approximately an 85–90% confluence, were obtained by trypsinization. After dissociation, the cells were mixed with Matrigel (Sigma-Aldrich) in a 1:1 ratio, obtaining a total volume of approximately 80 μL in a microtube. This mixture was collected using a 1 mL, 13 mm long insulin syringe and administered subcutaneously once on upper right flank of the anesthetized Nu/Nu strain mice.

The experiment began by inoculating 5 × 10^6^ cells per injection site and the appearance and development of the tumors were monitored daily. After two weeks, the tumors grew enough to begin treatments; tumors with approximate sizes of 18.5–102 mm^3^ were treated. Mice (n = 3) were divided into three experimental treatments: (a) mice treated with an intratumoural injection of ferrofluid of SPIONs, (b) mice treated with an intratumoural injection of ferrofluid of SPIONs plus magnetic hyperthermia, and (c) mice treated with intratumoural injection of ferrofluid of SPIONs plus electroporation, plus magnetic hyperthermia. Treatments were performed every 7 days until day 21. For irradiation, mice were exposed to a magnetic field with a frequency of 190 kHz and an amplitude of 50 mT for 20 min.

After the treatments were applied, the murine models were monitored using a UVP iBox small animal imaging system (Analytik-Jena, Upland, CA, USA), which allows non-invasive detection of fluorescent and bioluminescent indicators.

Tumors were measured with a Vernier caliper every 7 days. Observations with the UVP iBox Explorer^2^ were performed every seven days. Before placing the individuals inside the equipment, the tumors were measured and received the corresponding treatment. Tumor volume was calculated using the following equation: V = 0.5 × L × W^2^ [17], where V is the tumor volume, L is the length of the tumor, and W is the weight of the tumor. Before tumors exceeded 18 mm in length or width the specimens were sacrificed.

### 2.3. Statistical Analysis

Statistical analysis was performed by one-way ANOVA and Tukey test. A statistically significant difference was considered when the *p*-value was <0.05. The results are presented as the mean ± standard error of at least three replicates.

## 3. Results

### 3.1. Electroporation Enhances Cytotoxic Effect to Hyperthermia Caused by SPIONs on Melanoma-Derived Cell Lines

#### Effects of SPIONs and Combined Treatments on Cell Viability

**Figure 1:**
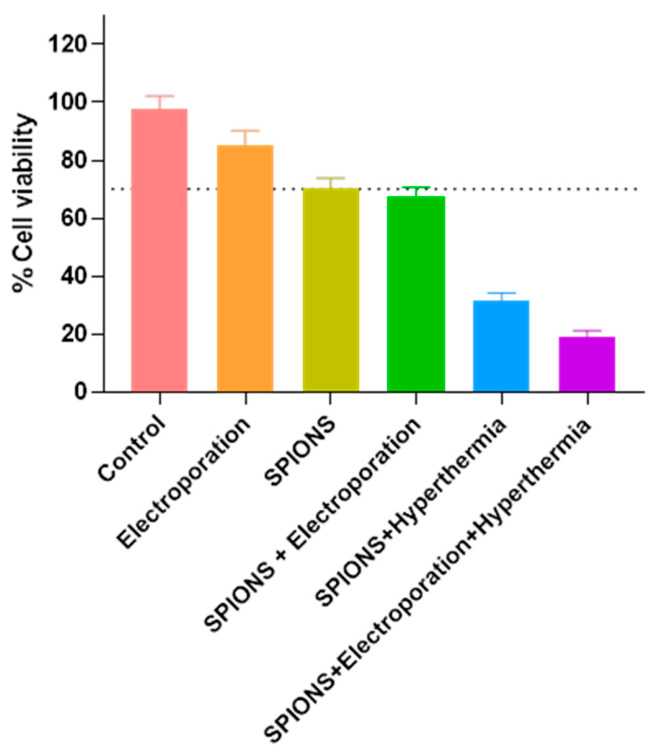
Cell viability (%) assessed by MTT assay 24 h after treatment. Bar graphs represent mean ± SD (N = 9 per group; three independent experiments, each performed in triplicate). The dashed horizontal line indicates the SPIONs-alone reference level (70.11%). Groups were compared using one-way ANOVA followed by Tukey’s HSD post hoc test. Asterisks indicate statistically significant differences relative to the SPIONs group Abbreviations: SPIONs, superparamagnetic iron oxide nanoparticles; EP, electroporation; Hyper, hyperthermia.

Determination of the optimal concentration of SPIONs for magnetic hyperthermia in melanoma-derived cell lines was performed as reported previously (Vizcarra Ramos et al., 2024) [13]. Cell viability was assessed by MTT assay 24 h post-treatment across six experimental groups (n = 3 independent experiments, each performed in triplicate; n = 9 observations per group). A one-way analysis of variance (ANOVA) revealed a statistically significant effect of treatment on cell viability (*F*(5, 48) = 630.81, *p* < 0.0001, η^2^ = 0.985), indicating that the experimental conditions collectively explained >99% of the variance in cell viability. Post hoc pairwise comparisons were conducted using the Tukey HSD test (*q* critical = 4.05, α = 0.05). Using SPIONs alone as the reference condition (70.11 ± 3.72%), three distinct response patterns were observed (Figure 1): (i) control and electroporation-alone groups exhibited significantly higher viability than SPIONs alone (control: 97.56 ± 4.67%, Δ = +27.44; electroporation: 85.44 ± 4.75%, Δ = +15.33; both *p* < 0.05, Tukey HSD), confirming that SPIONs alone produce a measurable baseline reduction in cell viability relative to untreated cells. (ii) SPIONs combined with electroporation (67.56 ± 3.13%, Δ = −2.56) did not differ significantly from SPIONs alone (*p* = n.s., Tukey HSD), suggesting that electroporation does not synergize with SPIONs to further reduce cell viability under the conditions tested. (iii) Hyperthermia-containing regimens caused profound and statistically significant reductions in cell viability relative to SPIONs alone. SPIONs + Hyperthermia reduced viability to 31.33 ± 2.87% (Δ = −38.78, −55%; *p* < 0.001), while the triple combination (SPIONs + Electroporation + Hyperthermia) achieved the lowest viability recorded: 19.22 ± 1.99% (Δ = −50.89, −73%; *p* < 0.001 vs. all other groups). These findings indicate that hyperthermia is the primary cytotoxic driver when combined with SPIONs, and that the addition of electroporation to this combination yields only marginal further reduction in viability.

### 3.2. In Vivo Treatment by Intratumoral Injection

**Figure 2:**
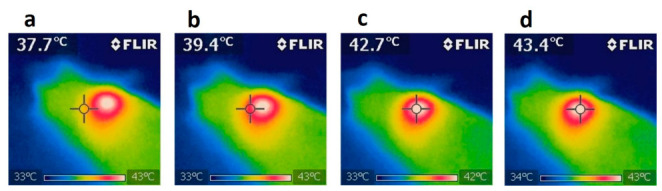
Thermographic images of temperature distributions during magnetic hyperthermia. (**a**) Initial thermal image approximately 37.7 ± 0.5 °C surrounding tumor tissue. (**b**) and (**c**) Midpoint of irradiation, temperature was approximately 39.4 ± 0.5 and 42.7 ± 0.5, respectively. (**d**) Final image after 10 min of irradiation, showing a peak temperature of 43.4 ± 0.5 °C.

For intratumoral treatment, SPIONs were administered every 7 days for up to 21 days of follow-up. In the electroporation and electroporation plus magnetic hyperthermia groups, a solution containing SPIONs (1 mg/mL) was administered at a final volume of 100 µL. A thermographic camera was used for real-time imaging to document temperature changes. The increase in temperature at the tumor site indicated activation of the SPIONs under magnetic stimulation, confirming effective and targeted photothermal conversion. Changes in tumor size were measured using a Vernier caliper and the IboxExplorer^2^/VisionWorksLS software version 8.1.2, generating color maps based on the intensity of fluorescence emitted by viable MV3/GFP cells. During the magnetic field irradiation process, the temperature of each mouse was continuously monitored and limited to 43–44 °C to prevent tissue overheating. The average temperature was maintained at 43.4 °C (Figure 2a–d). The highest temperature increase was concentrated primarily in the region where the SPIONs were located.

#### 3.2.1. Comparison of the Treatment in the Experimental Mice

The measurements shown in the previous graphs for each mouse were compared in a time-course graph, where changes in tumor size for each group can be observed from day 0 and on days 7, 14, and 21 in the in vivo experimental model.

**Figure 3:**
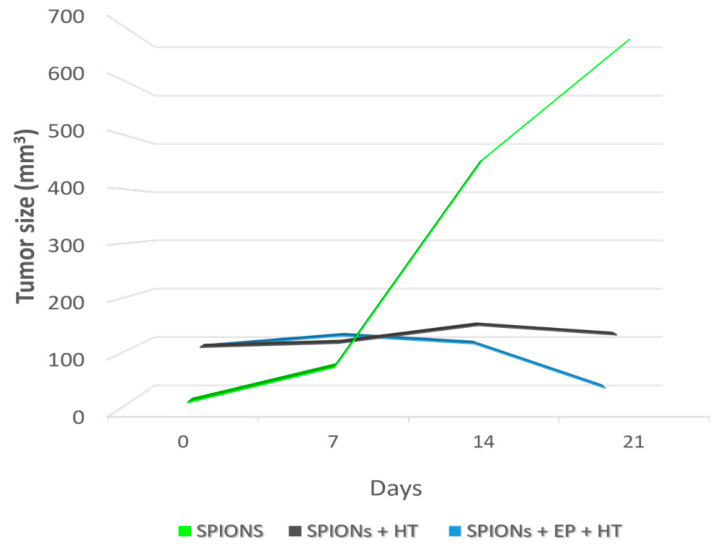
Effect of treatment on tumor growth. Tumor treated with only SPIONs reached a size of 665 mm^3^; on the other hand, the SPIONS + Hyperthermia (SPIONs + HT) tumor mouse showed a stabilization in size after treatment, however, again, reaching a tumor size of 126 mm^3^. The mice treated with SPIONs + Electroporation + Hyperthermia (SPIONs + EP + HT) showed a significant reduction in tumor size on day 21 and the tumor disappeared on day 28.

**Figure 4:**
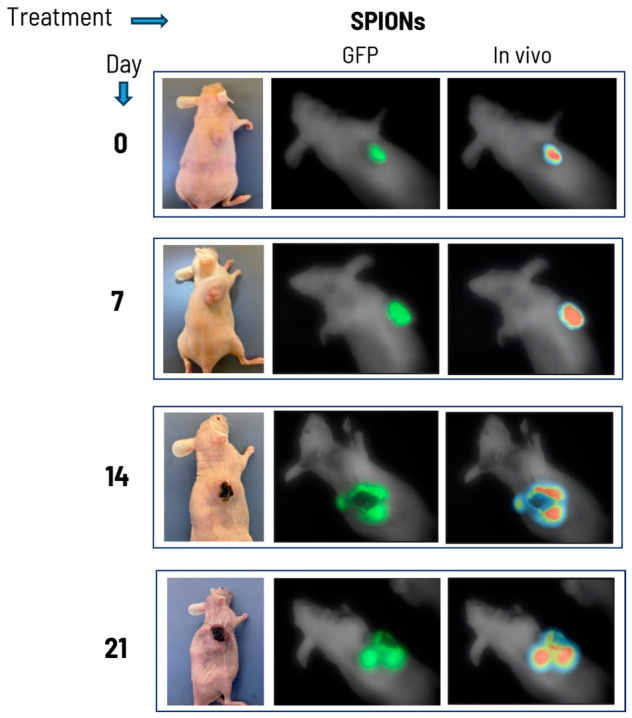
Effect of SPIONs on tumor growth in vivo. The change in tumor size and appearance at the beginning from day 0 and for days 7, 14, 21. GFP; tumor picture with Green Fluorescent Protein Filter (In vivo: tumor picture with “in vivo” shown place where are more tumoral cells (digital filter of the IboxExplorer^2^)).

**Figure 5:**
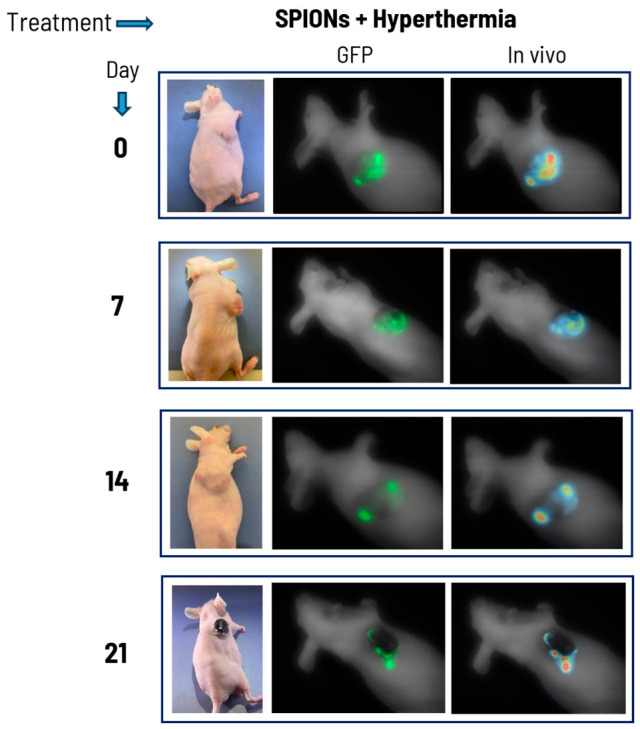
Effect of SPIONs plus magnetic hyperthermia on tumor growth in vivo. The change in tumor size is shown for days 7, 14, and 21. The reduction in tumor size and the fluorescence is evident on day 21. (In vivo: tumor picture with “in vivo” shown place where are more tumoral cells).

Mice treated with SPIONs alone showed uncontrolled tumor growth, increasing from 18.45 mm^3^ to a maximum size of 665 mm^3^ by day 21 (Figure 3 and Figure 4). In contrast, mice treated with SPIONs plus magnetic hyperthermia exhibited moderate tumor growth, suggesting an inhibitory effect, with tumor volumes of 110, 144, and 126 mm^3^ on days 7, 14, and 21, respectively (Figure 3 and Figure 5).

**Figure 6:**
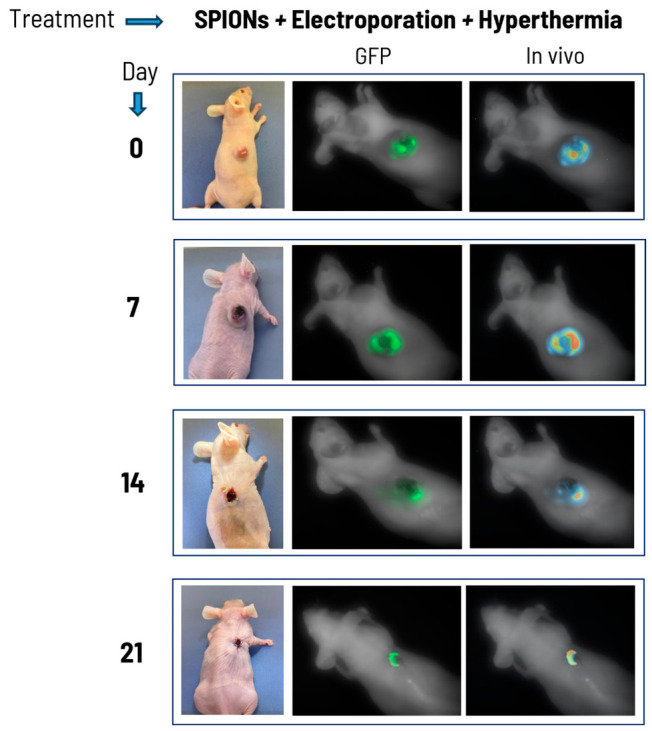
Effect of SPIONs plus magnetic hyperthermia on tumor growth in vivo. The change in tumor size is shown for days 7, 14, 21, and 28. Evidence of reduction in tumor size and complete elimination of the tumor at day 28. (In vivo: tumor picture with “in vivo” shown place where are more tumoral cells).

On the other hand, mice treated with SPIONs combined with electroporation and magnetic hyperthermia showed a significant reduction in tumor size at days 7 and 14, reaching the smallest size by day 21 (Figure 3 and Figure 6). No tumor mass or fluorescence signal was observed by day 28.

#### 3.2.2. Analysis on Fluorescent In Vivo Tumor Model

After MV3/GFP tumor cells were administrated, tumor evolution/involution for each mouse was documented photographically by using UVP iBox Explorer^2^ equipment (Analytik Jena Company, Jena, Germany) and VisionWorksLS Acquisition and Analysis Software version 8.1.2. This equipment made it possible to observe the fluorescence generated by the tumor cells that make up the tumor, allowing visualization of tumor development in response to treatment. The intensity of fluorescence correlates with cell viability. It was possible to determine the fluorescence of the tumor by generating MV3/GFP cells, which allowed us to visualize tumor development and evaluate its response to treatment in real time. It is important to note that the intensity of tumor fluorescence correlates with the viability of the tumor cells.

In the mouse treated with SPIONs, the fluorescence was evident and with a high signal; the tumor began with a size of 18.45 mm^3^, growing significantly to reach a size of 665 mm^3^ at 21 h (Figure 4). Immediately after the tumor was documented, the mouse was sacrificed.

In the mouse treated with SPIONs plus hyperthermia, fluorescence was evident and with a high fluorescence signal; the tumor started with a size of 102.8 mm^3^, reaching a maximum size of 144 mm^3^ at 14 days and reducing its size to 126.5 mm^3^ at 21 days. In comparison to the tumor in the mouse treated with SPIONs, the growth of this one was smaller (Figure 5). After the tumor was documented, the mouse was sacrificed.

In the mouse treated with SPIONs plus electroporation plus hyperthermia, fluorescence was evident and with a high signal; the size of the tumor started with a size of 90.04 mm^3^, growing at a slower rate than mice receiving the other treatments on days 7 and 14, and on day 21 the size was significantly reduced (Figure 6). On day 28 the tumor disappeared completely, this mouse was left in the animal facility for 30 more days and the tumor did not reappear and, finally, it was sacrificed.

## 4. Discussion

Electroporation is a physical method commonly used in electrochemotherapy to facilitate the uptake of large drugs such as bleomycin and cisplatin, which would otherwise possess limited access to the intracellular space. Recently, our research group has focused on implementing electroporation as a nanoparticle-delivery strategy, specifically for the superparamagnetic iron oxide nanoparticles (SPIONs) [13].

The present study hypothesizes that electroporation can enhance intracellular SPIONs uptake, potentially reducing the administered SPIONs concentration required to induce a significant cytotoxic effect, which is a critical consideration for clinical translation.

In this preliminary in vivo study, the SPIONs concentration used was 1 mg/mL, determined by Vizcarra-Ramos et al. [13] in a prostate cancer cell model, which was optimized for electroporation combination approaches. It is important to note that higher concentrations have been reported to induce cytotoxicity in various studies [18,19]. Although higher concentrations were not used in this preliminary study, the lower concentration reported by Vizcarra-Ramos et al. [13] was useful and had an important in vitro effect on skin cancer cells and on fluorescent melanoma tumors, indicating that electroporation could help to reduce the dosage of SPIONs; however, further research is needed.

One possible explanation is that electroporation enhances the endocytic uptake of nanoparticles. The SPIONs used in this preliminary in vivo study are 14 nm in size and normally enter the cell via clathrin-mediated endocytosis [13]; however, upon electroporation, a significant increase in magnetic hyperthermia cytotoxicity was observed, which may be due to a higher intracellular SPIONs concentration in combination with magnetic excitation. This is consistent with prior evidence in pancreatic adenocarcinoma cells, where electroporation increased intracellular iron oxide nanoparticle uptake in both in vitro and in vivo models [18], confirming that this strategy could enhance nanoparticle intracellular concentration. This is clinically significant, given that conventional magnetic hyperthermia requires delivering large doses of nanoparticles to tumors to achieve a significant antitumor effect.

When electroporation alone with SPIONs is applied, the cytotoxic effect of SPIONs alone slightly increases. When excited with a magnetic field, the cytotoxic effect significantly increases (in vitro results), which could be explained by the results reported in other studies, where it has been demonstrated that the nanoparticles internalized into tumor cells possess greater cytotoxicity when excited with an alternating magnetic field than an equivalent dose delivered by nanoparticles located in the extracellular medium [19], suggesting a localized effect that can be achieved through apoptosis, necroptosis or other mechanisms driven by small amounts of heat generated at thermosensitive intracellular sites [20].

Electroporation could be targeted directly to tumor tissue by properly positioning the electrodes, suggesting that if electroporation preferentially increases SPIONs uptake in tumor cells, magnetic hyperthermia will generate proportionally greater intracellular heating within tumor tissue than in adjacent healthy tissue, where SPIONs would remain mainly extracellular. Although this is a preliminary in vivo study and does not directly measure the effect on adjacent healthy tissue, the images above show that the only affected tissue was the tumor, suggesting that the combination of electroporation and magnetic hyperthermia could represent a strategy that concentrates the thermal cytotoxic effect within the tumor. The latter requires future studies that involve normal tissue controls.

Several important limitations must be acknowledged. First, there was no direct measurement of intracellular SPIONs uptake after electroporation and uptake was inferred only from MTT assays. Direct quantification using techniques such as inductively coupled plasma mass spectrometry or labeled nanoparticle imaging would provide more robust mechanistic evidence and should be explored in future studies. In addition, the preliminary nature of this study resulted in the omission of several important control groups. The inclusion of these controls would allow a more comprehensive assessment of the individual contribution of each treatment component and would strengthen the evaluation of potential synergistic effects. Therefore, the observed cytotoxic and antitumoral effects cannot be attributed exclusively to enhanced SPION uptake or to a synergistic interaction between electroporation and hyperthermia. Furthermore, the limited sample size in the in vivo experiments restricts the statistical power and generalizability of the findings. Finally, the potential reduction in damage to adjacent healthy tissue inferred from the combined approach was not directly evaluated in this study and remains a hypothesis to be addressed in future experimental designs.

On the other hand, multidrug resistance (MDR) has become a significant factor limiting the cure rate for cancer patients. While chemotherapy can induce apparent remission in tumors, in some cases, tumor cells develop resistance leading to disease relapse with catastrophic consequences. The evaluation of novel combination therapies without traditional drugs, such as those reported here, and their effectiveness, offer a potential solution to this MDR phenomenon in the not-too-distant future.

## 5. Conclusions

The preliminary in vivo results obtained in this study suggest that the combination of intratumoral administration of SPIONs with electroporation and magnetic hyperthermia significantly enhances the antitumoral effect compared to hyperthermia alone. The in vivo findings demonstrated notable antitumoral activity, with an initial delay in tumor growth during the first 14 days, followed by a pronounced therapeutic effect at days 21 and 28, culminating in complete tumor disappearance in the treated mouse.

These preliminary and brief results showed that combining electroporation plus magnetic hyperthermia significantly increases the cytotoxic in vitro assays and the antitumoral activity observed in these fluorescents tumors generated with MV3/GFP cells is an excellent model for in vivo evaluations.

## Data Availability

The original contributions presented in this study are included in the article. Further inquiries can be directed to the corresponding authors.
